# Academic Expectations of Stress Inventory: A Psychometric Evaluation of Validity and Reliability of the Persian Version

**DOI:** 10.3390/jpm11111208

**Published:** 2021-11-16

**Authors:** Mojtaba Habibi Asgarabad, Morteza Charkhabi, Zahra Fadaei, Julien S. Baker, Frederic Dutheil

**Affiliations:** 1 Health Promotion Research Centre, Iran University of Medical Sciences, Tehran 1445613111, Iran; habibi.m@iums.ac.ir; 2 Department of Health Psychology, School of Behavioral Sciences and Mental Health (Tehran Psychiatric Institute), Iran University of Medical Sciences, Tehran 1445613111, Iran; 3 Department of Psychology, Norwegian University of Science and Technology, 7491 Trondheim, Norway; 4 Center of Excellence in Cognitive Neuropsychology, Institute for Cognitive and Brain Sciences, Shahid Beheshti University, Tehran 1983963113, Iran; 5 Institute of Education, HSE University, 16 Potapovsky Pereulok Building 10, 101000 Moscow, Russia; 6 Family Research Institute, Shahid Beheshti University, Tehran 1983963113, Iran; 7 Center for Health and Exercise Science Research, Hong Kong Baptist University, Kowloon Tong 999077, Hong Kong; jsbaker@hkbu.edu.hk; 8 CNRS, LaPSCo, Physiological and Psychosocial Stress, CHU Clermont-Ferrand, University Hospital of Clermont-Ferrand, Preventive and Occupational Medicine, WittyFit, Université Clermont Auvergne, F-63000 Clermont-Ferrand, France; frederic.dutheil@uca.fr

**Keywords:** academic expectation, academic stress, validity, reliability, gifted and non-gifted students

## Abstract

This study aims to evaluate the psychometric properties of the Academic Expectations of Stress Inventory (AESI) in terms of validity and reliability measurements among Persian students. A total sample of 620 high-school students (*n*_female_ = 328, *n*_male_ = 292) was recruited to complete scales on academic expectations of stress, self-efficacy, and depression. The AESI was translated from English to Persian and its translation was further checked by three experts. We used a cross-sectional research design to collect data. The results approved the internal consistency, test–retest reliability, convergent, and construct validity of the ASEI. Additionally, confirmatory factor analysis confirmed the two-factor structure of the AESI, including the expectation of self and the expectations of parents/teachers. AESI was related to depression and self-efficacy in an empirically and theoretically expected direction. Moreover, configural and metric invariance were supported by gifted vs. non-gifted groups, but not scalar. No invariance was supported by gender groups. In conclusion, the psychometric properties of the Persian version of the AESI were confirmed to be used for educational, clinical, and research purposes in Iran.

## 1. Introduction

Academic stress is considered as the main source of stress among high-school students [1]. It is a prevalent circumstance that negatively influences the students’ psychological, educational, and physical states [2,3]. Academic stress is defined as the psycho-physiological response to stressful academic circumstances in which students are confronted during studying at schools or universities [4]. This stress is recognized as one of the main causes of malignant psychiatric statuses such as students’ suicide [5]. Academic stress is mostly experienced by students when academic performance (i.e., math performance) is assessed by a teacher. 

In collectivist countries, such as Iran, students may further experience academic stress because they have more social and family networks and their academic performance might be monitored or judged by surrounding people [6]. Although most studies on academic stress have been conducted in the U.S. [7,8], global statistics show that academic stress is rapidly growing in Asia and Middle Eastern countries including Korea [9] and India [10,11]. According to the National Crime Records Bureau [11], failing in exams has been the cause of 1.8% of suicides among Indian students.

These studies show that academic stress is still a dynamic, alive, and growing phenomenon influencing students all over the world; however, our investigation showed that it still has not been extensively studied in Iran. To better understand the academic stress in Iran, we need to add that education in Iran is divided into pre-university, which is 12 consecutive years a student needs to study before he/she obtains a diploma, and then the university education begins (4 years for a bachelor diploma, 2 years for a master diploma, and 3 to 4 years for a Ph.D.). The focus of this study is only on high-school students because this group of students in Iran is known to experience the highest academic stress in pre-university education. First, the GPA of high-school students has a direct statistical impact on the final score of entrance test which is calculated by computerized programs and by an operating organization annually. Thus, students are under huge pressure to obtain a high GPA during the nine months before they take the national entrance test (known as *Konkoor*). They need to attend classes and obtain excellent marks to increase their GPA. Students with a higher GPA may have a greater chance to enter the top universities. Second, they need to attend extra classes and read extra academic books (known as test books) to become familiar with the way that they can respond to the questions of the national entrance test, which will be held in two sections, general subjects (4 different subjects) and special subjects (4 different subjects), during a four-hour offline written test. If they miss this chance and do not perform well, then they cannot be admitted to a public university. It means that they must wait for the next national entrance test which will be held only once a year. This provides an epidemiologically suitable context to study the academic stress among Persian high-school students. Additionally, high-schools in Iran are divided into two types: public schools and Sampad schools. Public schools normally include students that pass their exams and move from a lower grade to an upper grade, and we considered them as non-gifted in the current study. Sampad schools are those that students are required to take an entrance exam before they can enter them. Sampad schools normally include gifted students.

## 2. A Review on Academic Stress and Health

The majority of previous studies have introduced high-school students as the group more exposed to academic stress [12]. Academic stress occurs when students encounter problems in adapting to academic environments such as schools or universities which may affect their academic performance. According to available statistics, about 10% to 30% of American high-school students experience different levels of stress during their education [13]. Research shows that academic stress can influence a wide range of psychological outcomes. It has been reported that academic stress is the main cause of depression, suicide, mental and behavioral disorders, and anxiety among students [14,15].

According to the American College Health Association [16], academic stress was reported as the basic predicament of college students, resulting in lower academic performance (i.e., failure to obtain minimum marks or complete a given course) for 32% of the students. Additionally, academic stress was found to have a direct negative impact on students’ self-esteem [17] and school dropout rates [18]. Results of a study on 872 adolescent school students in Hong Kong demonstrated a positive relationship between parental expectations and students’ academic stress and depression [19]. Besides, factors such as school reputation may further intensify the harmful impacts of academic stress, and as such, students enrolled in schools with higher academic standards may even report higher levels of academic stress. For example, a cross-cultural study including Singaporean and Canadian adolescents’ samples showed that Singaporean adolescents reported a higher level of academic stress than Canadians, especially in expectations of self [6]. Another example can be seen when students enter a new academic environment. A relevant study shows that these students will confront abundant requirements such as time pressure, homesickness, matching with college members, and anxiety [20].

Multiple reasons have been suggested as the cause of academic stress. Parents’ high expectations are considered as one of the major causes of academic stress in adolescents and were found to be negatively associated with self-esteem in East Asian American students [21]. Other studies have also suggested factors such as the great number of assignments and a short time available for completing the assignments [22], commitment pressure to complete school assignments [23], lack of time management in school-related activities [24,25], and financial problems [26] that may produce or intensify the academic stress, and in extreme cases lead to an increased level of adrenalin hormone, heartbeat, significant neurohormonal changes in the hypothalamic-pituitary-adrenocortical axis, and substantial dysfunctions in the immune system of the body [27,28].

Students may experience different levels of academic stress because they may use different coping strategies to cope/deal with the academic stressors [25,29]. This may be the reason why the negative consequences of academic stress may be seen in physiological reactions or behavioral reactions, such as chronic lack of sleep [24,30], increased alcohol consumption [5], and poor eating patterns [31]. These consequences show that academic stress may lead to serious negative outcomes and needs to be identified and controlled before it results in these outcomes.

There are two types of academic stress that students may confront: subjective and acculturative academic stress. Subjective academic stress refers to the kind of stress that can influence students’ behaviors, cognitive, and physiological states differently. That is why when individuals deal with this kind of stress, they may experience less or more mental pressure, which is supported by the diathetes theory [32]. According to this theory, some individuals are more vulnerable, psychologically, and physiologically, against a perceived stressor than others, and subsequently experience more detrimental outcomes [32,33]. Acculturative academic stress is felt when students meet new individuals, new cultures, new languages, and a new lifestyle in a new school. Indeed, they may appraise the new situation as a demanding situation in which they struggle to cope [34]. Those who cannot cope with this situation may experience psychosomatic disorders such as sleep disorders, headaches, and body dysfunctions, while others may express this stress in the form of depression and anxiety [35]. An interesting study showed that international students in American universities, regardless of their language and culture, report more adjustment issues than locals [36,37,38]. Perhaps this is because the students may feel more relaxed when they perceive that their school culture is closer to their own culture. This is consistent with the study of Ang [6], who found that Canadian students reported lower academic stress than their Singaporean peers at a Canadian university. The academic expectation of stress is a kind of subjective stress that a student may experience when they cannot cope/deal with the demands or expectations in a given situation. We assume that mental pressure can influence both the self-efficacy and depression of students.

## 3. Convergent and Construct Validity of AESI

In this study, we use depression and self-efficacy as two well-known correlates of academic stress to test the convergent and construct validity of the AESI. Previous studies have reported that there is a positive association between academic problems and depressive mood [39,40,41]. This is also related to previous findings that show that poor academic performance is associated with depression and developing depressed feelings among students [41,42]. One reason is that children who face academic difficulties are shamed or humiliated by parents, peers, and teachers, and the repeated exposure to negative feedback inhibits them to develop positive self-schema and results in depression [43]. The depression associated with the bad performance of students in exams has been found to be related to suicide among students [11,44,45]. Students with high academic stress who show depression symptoms have difficulties with class concentration and fear of failure in exams [46] and are more likely to become involved in health risk behaviors such as alcoholism, drug use, risky unprotected sexual behaviors, and eating and sleep disorders [5]. Thus, depression can be used as a suitable variable to test the convergent validity of AESI.

Although researchers have reported a positive association between self-efficacy and academic achievement [47,48], the link between academic stress and self-efficacy was found to be negative [49,50]. This may be because when students believe that they can fulfill their academic activities, known as academic self-efficacy [48,51], they are less likely to feel stressed because they can meet their academic goals [52,53]. On the contrary, students with low self-efficacy are more likely to experience academic stress because of the lack of belief [50]. Thus, self-efficacy can be used as a suitable variable to test the construct validity of AESI.

## 4. Academic Expectations of Stress Inventory

The Academic Expectations of Stress Inventory (AESI) is an applied research tool for both scientists and practitioners to measure the specific role of students’ expectations and their supervisors’ expectations (parents/teachers) in producing or exacerbating academic stress. The initial version of AESI developed by Ang and Huan [44] consisted of 13 items and 2 components of expectations of self and expectations of others (parents/teachers). Higher scores indicate higher perceived academic stress. Ang and Huan [44] reported Cronbach’s alphas of 0.87, 0.84, and 0.83 for the total, self-expectations, and parents/teachers’ expectations components, respectively. Moreover, they examined the 2-week test–rest reliability and reported coefficients of the three aforementioned components as follows, respectively: 0.79, 0.85, and 0.77. Additionally, the results of exploratory factor analysis on a sample of 721 Asian adolescents suggested a nine-item scale consisting of two components: teachers and parents’ expectations including five items, and expectations of self, including four items [44]. A similar study conducted in Taiwan included students of 10 universities to validate the AESI. The overall Cronbach’s alpha of academic expectations of stress was obtained as 0.90. Additionally, the validity of this inventory was assessed by K-Pearson’s correlation tested on seven factors of the AESI, including teacher stress, results’ stress, test stress, group study stress, peer stress, time management stress, and self-induced stress, which were from 0.63 to 0.85 [41]. Besides, various researchers used different scales to test the validity of this scale (FNE-Brief, CDI-Short, and RCMAS, reported by previous researchers [54,55,56]).

## 5. Academic Expectation of Stress and Academic Giftedness

We assume that there might be a difference between the degree to which gifted and non-gifted students are influenced by academic expectations. This is because studies show that gifted children, due to their unique characteristics, are more likely to suffer from academic stressors [57,58]. Academically gifted students may experience higher levels of stress and anxiety due to their higher intellectual abilities, which drive them to be more perfectionist, sensitive, and to have socialization problems [59,60] or lower life satisfaction [57]. The most probable troubles they confront are dealing with the intensification of their homework, selecting an appropriate field of research and study, tolerating extra weariness, their peers’ negative way of thinking about them, and difficulty on how to understand others (as they are often introverted), which may cause the gifted students to experience academic stress [61]. As such, we expected to find greater academic stress among Iranian gifted students than their non-gifted peers.

## 6. Academic Expectations of Stress and Gender

Gender difference is assumed to influence the way students perceive and react to academic stress [7]. While female students show their emotions to stress overtly, it seems the male students prefer not to reveal them explicitly and use more problem-solving strategies [62]. This is consistent with the study of Alsulami [63], who showed that females’ perception of academic stress was significantly higher than that of males and the perception of academic stress among females was more affective and physical, respectively. Additionally, a similar study showed a significant difference between males’ and females’ scores in the Academic Expectations Stress Inventory (*f = 0*.954, *p* < 0.05), while women (*M* = 12.29, *SD* = 7.39) obtained higher meaningful scores in the fear of failure dimension of AESI (*f* = 5.207, *p* < 0.05) than men (*M* = 10.53, *SD* = 6.50) [45]. Although the majority of studies revealed that female students show higher academic stress than their male peers [64], a few studies found that male and female students perceive academic stress similarly [18]. Additionally, it should be noted that the causes of academic stress may be slightly different between male and female students. For example, some studies showed that while female students report more academic stress facing stressors such as worries and anxiety about progress at school, male students report more academic stress when they need to deal with parents and teachers’ demands [65,66]. These findings show that we should expect to find a significant difference between Persian male and female students on AESI as well.

## 7. Method

### 7.1. Participants

It should be noted that the public schools in Iran are divided into gifted schools, known as Sampad, and non-gifted schools. In this study, we had access to three gifted high-schools and three non-gifted high-schools. To enroll in a gifted high-school, a student needs to pass a highly competitive entrance test, including all school subjects plus an intelligence test. The number of gifted high-schools is less than the non-gifted high-schools in each city of Iran, and only a small portion of students have the chance to gain a seat in a gifted school.

We recruited 620 high-school students including academically gifted students (*n* = 312; *M_age_* = 15.93, *SD* = 1.37; 53.2% were female) and non-gifted students (*n* = 308; *M_age_* = 15.95, *SD* = 1.38; 52.6% were female) using multi-stage cluster sampling. The inclusion criteria for recruiting students were (1) enrolled in a high-school, (2) aged between 14 and 18, and (3) being single (not being in a relationship). All students were enrolled full-time and were from different educational levels of high-school. The students were recruited from six public high-schools in the city of Tehran (three feminine high-schools and three masculine high-schools).

The distributions of responses of non-gifted students were as follows: 114 (32.4%) high-school first-year students, 34 (11%) second year, 70 (22.7%) third year, 1 (0.3%) fourth year, and 89 (28.9%) did not answer this question. The same distributions in the gifted group were as follows: 90 (28.8%) high-school first-year students, 70 (22.4%) second year, 20 (6.4%) third year, 49 (15.7%) fourth year and 83 (26.6%) did not answer this question. We randomly chose 30 students (50% female) from the non-gifted group (*n* = 308) and 30 students (50% female) from the gifted group (*n* = 312) for the test–retest validity. In total, a combination of 60 students participated in the pilot part of this study. These students agreed to voluntarily participate in the retest study as well. The time interval between the test and retest studies was four weeks.

### 7.2. Procedure

This study was submitted to the ethics board of the Iran University of Medical Science located in Tehran, Iran, and conducted under the supervision of this university. The research team contacted a list of public and Sampad high-schools in Tehran to negotiate about the possibility of conducting this study in these schools. Six high-schools out of ten (whom we contacted) agreed to participate in this study subject with the voluntary participation of students and a signed consent letter from students’ parents: 700 students were preliminarily invited to participate, and 620 accepted and met the conditions to participate (response rate = 89%). The research team distributed the self-report survey among the students identified as gifted and non-gifted groups. Students were informed that they could ask any questions during the study and they were informed about their rights to withdraw from the study in any step if they wanted to. Students were asked to write their student identification number to match each survey with school grades in pretest and test steps.

The AESI was translated into Persian by an academic team including a psychiatrist and two health psychologists. All items were checked one by one by all the team members to ensure that the translated version conveyed the intended meanings in Persian. Then, the Persian version of the scale was back-translated to English by two linguists. Following that, a bilingual individual with expertise in the English language compared the back-translated and original versions of the scale to remove the inconsistencies between the two versions. The current Persian version of the AESI was piloted with 60 participants (50% non-gifted and 50% gifted) who confirmed a comprehensive understanding of the scale, and they were asked to rate the readability and clarity of the Persian language items individually on a 0 (not understandable) to 5 (completely understandable) Likert scale. The most common rating by participants was “completely understandable” (response option number five), with this response option endorsed at 95% or higher across all items. Thus, participants’ responses indicated no need for item revision. As the scale had initially been developed in another country and in this study we only aimed to test its psychometric properties, therefore, there was no reason to run an exploratory factor analysis to justify the components and items. Instead, we used confirmatory factor analysis to confirm the existence of components and items in the new context.

### 7.3. Measures

Academic Expectation of Stress Inventory (AESI): This inventory was developed by Ang and Huan [44] to measure academic stress among adolescent students (11 to 18 years). This scale consists of 9 items, and scores are recorded on a 5-point Likert scale (1 = never, 2 = rarely, 3 = sometimes, 4 = often, and 5 = always). AESI includes the two following components: expectations of self (4 items), and parent and teacher expectations (5 items). Higher scores indicate higher perceived academic stress. Ang and Huan [44] reported the total internal reliability in terms of Cronbach’s alpha for the whole scale, expectations of self, and expectations of parents/teachers as 0.89, 0.84, and 0.85, respectively. They also reported 0.875 for test–retest reliability of the AESI (with a two-week interval). The Cronbach’s alpha of teacher/parents’ expectations and expectation of self in the current study were 0.839 and 0.826, respectively. The overall Cronbach’s alpha of this scale was 0.890 in this study.

Children’s Depression Inventory (CDI): This scale was developed by Kovacs [54] to measure both child and adolescent depressive symptoms. This scale has 27 items that can be allocated to the following 5 components: (1) negative mood, (2) interpersonal problems, (3) ineffectiveness, (4) anhedonia, and (5) negative self-esteem, which include both feelings and behaviors (e.g., low mood, trouble sleeping, relationship and school problems). Items are rated on a three-point scale from 0 to 2, graded in severity. The CDI has extensive support for its psychometric properties [67]. Results of an adapted CDI with 12- to 19-year-old Iranian adolescents revealed test–retest reliability and internal consistency of 0.82 and 0.83 [67]. In the current study, the Cronbach’s alpha of the scale was 0.85.

Self-Efficacy Questionnaire for Child (SEQ-C): This scale was initially developed by Muris [68] and it contains 21 items that can be allocated into the following 3 components: (1) academic self-efficacy, (2) social self-efficacy, and (3) emotional self-efficacy. Each item was rated on a 5-point Likert scale ranging from 1 (not at all) to 5 (very well). In a validation study of the SEQ-C, the Cronbach’s alphas for the 21-item version of the scale, social self-efficacy, academic self-efficacy, and emotional self-efficacy were 0.90, 0.82, 0.84, and 0.86, respectively [69]. Results of an adapted SEQ-C with 14- to 19-year-old Iranian adolescents approved the original three-factor structure: social, academic, and emotional self-efficacy, and revealed that inter-item correlation ranged from 0.68 (social self-efficacy) to 0.79 (emotional self-efficacy), and the test–retest reliability for total scores of the self-efficacy, social, academic, and emotional subscales were 0.80, 0.68, 0.77, and 0.79, respectively [70].

## 8. Data Screening and Statistical Strategy

Due to the fact that the missing data ratio was less than the critical rate (1.8%), list-wise deletion with no imputation of data was used [71]. No transformation was performed because of the large sample size [69]. Regarding the non-normality of data and large sample size, the weighed least squares (WLS) estimation method was employed as it is less sensitive to the lack of normality of ordinal data. None of the cases met the criteria to be classed as a univariate and multivariate outlier. In addition, we tested the reliability of the study measures using Cronbach’s alpha and test–retest reliability. The criterion validity, including convergent and discriminant validity, was evaluated by examining the correlations between the AESI scores with self-efficacy and the child depression inventory.

The structural equation modeling, using AMOS, version 26 [72], was used to evaluate the measurement model fit using confirmatory factor analysis (CFA [73]) [67,68,69,70,71,72,73]. Confirmatory factor analysis offers a variety of statistical tests and indices designed to assess the goodness-of-fit of identified models [74]. Consistent with Edinger and Wohlgemuth’s model [75], all standardized factor loadings were greater than 0.40. The model fit is assessed based on the following indices [76]: *χ*^2^, which is very sensitive to sample size and non-normality of the data—a non-significant *χ*^2^ implies the goodness-of-fit of the model to the data, RMSEA, which is a fit measure based on population error of approximation—a RMSEA value below 0.08 indicates a good fit and values below 0.10 represent reasonable errors of approximation in the population, CFI, which is an incremental fit index and represents the proportionate improvement in the model fit by comparing the target model with a baseline model, NFI = normed fit index, and TLI = Tucker–Lewis coefficient. For the present study, goodness-of-fit was evaluated using the following statistics: NFI and CFI, both higher than 0.90, normal chi-square (NC: less than 2 [77]), and SRMR and RMSEA, with a 90% confidence interval (<0.08 [78]). The optimal levels of all the fit indices [74] are summarized here as: GFI > 0.9, NFI > 0.90, TLI > 0.90, CFI > 0.90, RMSR < 0.08, 3 > *χ*^2^/df < 2, and RMSEA < 0.05, with a 90% confidence interval.

## 9. Results

Table 1 demonstrates means and standard deviations and the correlation matrix between research variables. As the table shows, AESI was negatively associated with self-efficacy (*r* = −0.638, *p* = 0.01). Additionally, there was the same significant negative association between the three components of self-efficacy and AESI (see Table 1). This verifies the construct validity of this scale in the present study. Additionally, according to Table 1, AESI is positively associated with depression (*r* = 0.787, *p* = 0.01), which confirms the convergent validity of this scale in this study.

We also divided the total score of each component by the number of its items to compare the components across gender. The mean score and standard deviation in the self-expectation component were 3.07 and 1.13 for male participants and 3.26 and 1.16 for female participants. Additionally, the mean score and standard deviation for the teachers/parents’ expectations component were 3.08 and 1.04 for male participants and 3.02 and 1.06 for female participants. The independent sample *t*-test suggested a statistically significant difference between male and female participants for the self-expectation component (*F* = 0.081, *df* = 578, *t* = −1.97, *sig* = 0.049). No statistically significant difference between male and female participants was found for the teacher/parents’ expectations component.

Confirmatory factor analysis (CFA) was conducted using structural equation modeling (AMOS, version 26) to examine the factorial structure of this scale. We examined the fit of four nested models to find the most parsimonious model. The results are presented in Table 2. Model 1 specified a two-factor model in which all 9 items were loaded on 2 factors of parents/teachers’ expectations and self-expectation. As Table 2 shows, the fit indices were out of the acceptable range for the model indices. After we checked the modification indices, the system revealed that two error residuals have a high correlation and needed to be covaried (items 8 and 9). In Model 2, we covaried the two errors and the fit indices improved. However, some model fit indices still were problematic. We checked the modification indices again, and based on the list of high-error residuals, we covaried errors of items 1 and 5 in Model 3. Although the fit indices improved in Model 3, checking the modification indices, we found there are still two more error residuals that when we covaried (items 5 and 3; items 5 and 4) all the model fit indices improved in Model 4. In the modification indices of Model 4, no more unusual covariance was observed, and as the fit indices were matched with the acceptable range, therefore, we chose this model as the final model.

Generally, decisions to correlate error variance rely on high common variance and whether their association significantly improves the model fit indices. More specifically, correlating error terms with high covariance can enhance the reliability of a latent construct, as measured via goodness of fit indices [79]. It should be noted that correlating error variances should be performed skeptically, with the consideration that correlating error variances does not hurt the fit indices of parsimonious aspects of the model [79]. The results were replicated with the JASP program (https://jasp-stats.org/ (accessed on 4 November 2021)) and were identical. The final model is matched with the model proposed by Ang and Huan [44]. Table 3 represents all the factor loadings for the four tested models. In the final model, 5 items for the parents/teachers’ expectations and 4 items for self-expectation were confirmed. The factor loadings in the final model ranged from 0.674 to 0.828.

The test–retest reliability of the total score of the academic expectation of stress and its two components (expectations of parents/teachers and expectations of self) for a 4-week interval was 0.78, 0.77, and 0.72, respectively

## 10. Measurement Invariance of AESI over Gender and Gifted/Non-Gifted Groups

We used the measurement invariance as a statistical method to indicate that the present inventory measures the same construct in the same way across studied groups. This basically includes the three followings’ steps: (1) configural invariance, which is the equivalence of models across groups, (2) metric invariance, which is the equivalence of factor loadings across groups, and (3) scalar invariance, which considers the equivalence of items’ intercepts across groups.

We tested the measurement invariance of AESI across gifted vs. non-gifted groups and male vs. female groups. The results are presented in Table 4. We tested the measurement invariance of AESI at the baseline model (configural), factor loadings (metric), and intercepts (scalar) levels. We used these three measurement invariance parameters to test the invariance of items across both gifted/non-gifted and male/female groups. As shown in Table 4, the final model, across gifted and non-gifted groups, in configural and metric measurements was invariant, but not in scalar measurement. Additionally, as shown in Table 4, the final model was not invariant across gender in configural, metric, and scalar measurements.

## 11. Discussion

The purpose of this study was to examine the psychometric properties of the AESI in terms of validity and reliability among Persian students. To test the convergent and discriminant validity of the AESI, we needed to identify and use two psychological constructs that have been positively and negatively associated with the AESI in previous studies. In doing so, self-efficacy had been reported to be negatively related to AESI [80] and child depression was positively associated with AESI [44] in previous studies. Our findings replicated the previous findings on the negative association between AESI and self-efficacy. Thus, the negative association supported the construct validity of this scale in Iran. Furthermore, our findings supported the positive association between AESI and depression, and because of the replication, we provided support for the convergent validity of the scale in Iran. These results are consistent with previous studies [44,80].

Using confirmatory factor analysis and considering the original model proposed by Ang and Huan [44], we tested four nested models to find the model with the most parsimonious fit indices (see Table 2). The difference between the competing models was about the size and number of errors of covariance that modification fit indices of the AMOS program automatically suggested to us. Model 4 indicated the most parsimonious fit indices and therefore was selected as the final model. This model included two components of parents/teachers’ expectations (5 items) and self-expectation (4 items). Although we covaried some error residuals, no item was discarded from the final model because the factor loading of all items was in an acceptable range (<0.40).

Comparing the two components of AESI, we found that the mean score of teacher/parents’ expectation was higher than the mean score of self-expectation. The finding on the average score of both components of AESI is consistent with previous studies. For example, in a similar study, Caucasian participants reported that adolescents’ perceptions of their parents’ educational expectations were higher than their educational expectations [81]. This also can be supported by the study of Ang [6], who tested two groups of Canadian and Singaporean students on AESI, and regardless of their nationality, they similarly reported the highest internal reliability for the total score, expectations of parents/teachers, and expectations of self in both groups [82]. It shows that cultural background and ethnicity cannot significantly influence the perception of AESI. Perhaps an explanation could be that Asian adolescents, especially gifted students, are assumed to be more sensitive to others’ judgments [83]. However, students with more positive interactions with their teachers are found to have lower levels of academic stress [84].

To test the extent to which the final model may be stable over gender (male and females) or groups (gifted and non-gifted), we used measurement invariance analysis. As can be seen in Table 4, our findings revealed that it is more likely that the final model is stable across gifted and non-gifted students than across gender. In other words, there was no difference in factor loadings of this model over gifted and non-gifted groups of students; however, the difference appeared at the scalar level, which means the perceptions of groups at the intercept level are not the same. Measurement invariance over male and female students at both metric and scalar levels was not invariant, which means that gender can play a role in the way students may perceive items of the AESI differently. Although it is out of the scope of this study to further investigate the role of gender, we recommend researchers take this into consideration.

We also compared the AESI across male and female students, and the between-subject effects showed that female students rated higher in the total score of AESI and its two components than male students. These results are consistent with Hoferichter and Raufelder’s study [2], which showed that female students’ scores were higher than male students in both emotional and physiological manifestations of academic stress. One explanation for this finding would perhaps be related to their physiological structure, which may put female students in a vulnerable position to experience more symptoms of stress [85]. A second explanation may be related to different coping strategies male and female students choose to deal with this stress. As noted earlier, male participants were found to be significantly different in their level of adjustment to academic stress than female participants [46]. Although we found that gender may influence the degree to which students perceive academic stress and subsequently the degree to which they experience the consequences of this stress, some studies do not support our findings [44].

Although in the original study [44], a 2-week interval was used to measure the test–retest reliability, and we used a 4-week interval to measure the test–retest reliability of AESI, the size of test–retest reliability in this study is consistent with the original study. The finding suggests that the test may be stable over a longer time and does not appear to be unnecessarily prone to temporal errors.

This study has some limitations. First, the assessment instruments we applied to collect data are self-administrated. Although the research team provided students with precise and clear instructions to respond to the questions, due to social desirability, some may have responded inaccurately. This can be controlled in future studies by using more diverse methods of data collection. Second, in this study, we only used convergent and construct validities to verify the validated version of this scale in Iran. Future studies may use more diverse types of validity measurements to re-verify the validity of this scale.

## 12. Conclusions

This study not only replicated some of the previous findings on the extent to which the AESI is associated with depression and self-efficacy in a non-western country, but also confirmed the validity and reliability of the AESI scale in Iran. This validated inventory can be used for the following purposes in Iran: First, this inventory can be used by school psychologists to identify the extent to which the detrimental educational expectation of teachers/parents may influence students’ depression or poor self-efficacy. The vulnerable students can be identified and be provided with social support or psychological interventions. Second, school managers may use this information to resolve the students’ problems through speaking with the students’ families and parents. Thus, they can act as a more efficient mediator between students and their parents. Third, this inventory can also be used as a screening instrument to periodically monitor the academic stress of students from before and following help-seeking. Fourth, clinical psychologists will be able to make more correct and precise diagnoses on the type and severity of the academic stress the students may experience and faster detect and explain the cause of other clinical disorders, such as depression or poor self-efficacy in school. Fifth, this inventory can be used to compare the epidemic rate of AESI in Iranian schools for international research purposes and subsequently for using similar intervention programs that may be developed in other countries based on using this scale.

## Figures and Tables

**Table 1:** Correlation matrix of research variables in the current study (*n* = 620).

	Variables	Items	*M*	*SD*	1	2	3	4	5	6	7
1	**Academic stress**	9	27.94	9.07	-						
2	Parents/Teachers ^1^	5	15.26	5.27	0.925 **	-					
3	Self-expectation ^1^	4	12.70	4.61	0.901 **	0.668 **	-				
4	**Self-efficacy**	21	54.28	15.35	−0.638 **	−0.589 **	−0.557 **	-			
5	Social ^2^	7	16.93	6.43	−0.614 **	−0.540 **	−0.571 **	0.842 **	-		
6	Academic ^2^	7	18.29	6.16	−0.548 **	−0.517 **	−0.464 **	0.869 **	0.619 **	-	
7	Emotional ^2^	7	19.40	5.75	−0.484 **	−0.459 **	−0.407 **	0.827 **	0.513 **	0.597 **	-
8	**Depression**	27	26.63	7.93	0.787 **	0.695 **	0.720 **	−0.605 **	−0.591 **	−0.473 **	−0.389 **

Notes: ^1^: refers to components of academic expectation of stress; ^2^: refers to components of depression; ** *p* < 0.01.

**Table 2:** Results of confirmatory factor analysis on AESI items (*n* = 620).

Model	χ*^2^*	*df*	RMSEA	CFI	NFI	TLI	SRMR	ECVI
M_1_	225.04	26	0.111	0.922	0.913	0.892	0.049	0.424
M_2_	183.59	25	0.101	0.938	0.929	0.911	0.045	0.361
M_3_	150.85	24	0.092	0.950	0.942	0.926	0.041	0.311
M_4_	94.92	22	0.073	0.972	0.963	0.953	0.032	0.257

Notes: χ^2^ = Chi-Square test for univariate normality. *df* = degree of freedom; RMSEA = root mean square error of approximation; CFI = comparative fit index; NFI = normalized fit index; TLI = Tucker–Lewis coefficient; SRMR = standardized root mean square residual; ECVI = Expected Cross-Validation Index.

**Table 3:** Results of factor loading of AESI items in the four tested models (*n* = 620).

		Model 1	Model 2	Model 3	Model 4
Expectations of Parents/Teachers	1. I blame myself when I cannot live up to my parents’ expectations of me.	0.656	0.657	0.681	0.685
	2. I feel I have disappointed my teacher when I do badly in school.	0.777	0.779	0.781	0.743
	3. I feel I have disappointed my parents when I do poorly in school.	0.780	0.779	0.766	0.802
	4. I feel stressed when I know my parents are disappointed in my exam grades.	0.697	0.695	0.677	0.712
	5. I feel lousy when I cannot live up to my teacher’s expectations.	0.690	0.691	0.722	0.828
Expectation of Self	6. I feel stressed when I do not live up to my own standards.	0.755	0.778	0.778	0.783
	7. When I fail to live up to my own expectations, I feel I am not good enough.	0.729	0.745	0.747	0.736
	8. I usually cannot sleep and worry when I cannot meet the goals I set for myself.	0.734	0.679	0.681	0.687
	9. When I do not do as well as I could have in an examination or test, I feel stressed.	0.730	0.674	0.671	0.674

**Table 4:** Results of measurement invariance analysis on the final model of AESI items (*n* = 620).

Group	Invariance	χ^2^	*df*	RMSEA	CFI	ΔCFI	NFI	TLI
Gifted/non-gifted	Configural	168.65	44	0.068	0.953	-	0.938	0.923
	Metric	174.65	53	0.061	0.954	−0.001	0.936	0.937
	Scalar	253.70	58	0.074	0.926	−0.028	0.907	0.908
Male/Female	Configural	135.80	44	0.058	0.965	−0.039	0.949	0.942
	Metric	153.78	53	0.055	0.961	0.004	0.942	0.947
	Scalar	176.12	58	0.057	0.955	0.006	0.934	0.944

Notes: χ^2^ = Chi-Square test for univariate normality. *df* = degree of freedom; RMSEA = root mean square error of approximation; CFI = comparative fit index; NFI = normed fit index; TLI = Tucker–Lewis coefficient.

## Data Availability

Data is available upon request at this email contact: habibi.m@iums.ac.ir.
